# Patient-reported symptom and urgent healthcare use in neuroendocrine tumors

**DOI:** 10.1530/EO-25-0050

**Published:** 2026-01-19

**Authors:** Julie Hallet, Christopher Noel, Rinku Sutradhar, Katrina Duncan, Calvin Law, Simron Singh, Sten Myrehaug, Natalie Coburn, Wing C Chan, Anna Gombay, Antoine Eskander

**Affiliations:** ^1^ Temerty Faculty of Medicine, University of Toronto, Toronto, Ontario, Canada; ^2^ ICES, Toronto, Ontario, Canada; ^3^ Clinical Evaluative Sciences, Sunnybrook Research Institute, Toronto, Ontario, Canada; ^4^ Susan Leslie Clinic for Neuroendocrine Tumors – Odette Cancer Centre, Sunnybrook Health Sciences Centre, Toronto, Ontario, Canada

**Keywords:** neuroendocrine, urgent care, patient-reported, carcinoid

## Abstract

**Background:**

Patients with neuroendocrine tumors (NETs) have a high burden of symptoms persisting over years after diagnosis. Patient-reported outcome measures are routinely screened for in oncology practice but seldom used for interventions. Further information about how they are linked to outcomes could improve their use. We examined the association between patient-reported symptoms and urgent healthcare use after NET diagnosis.

**Patients and methods:**

We conducted a population-level retrospective cohort study of adults diagnosed with NETs over 2010–2019. The exposure was Edmonton Symptom Assessment System (ESAS) scores within 2 years of diagnosis. The outcome was urgent healthcare utilization (emergency department visits and/or unplanned hospital admission) in 14 days after ESAS assessment. Logistic regression models examined the association between ESAS scores and the outcome.

**Results:**

A total of 4,278 patients completed 19,612 ESAS assessments. Each 1-point increment in drowsiness (OR: 1.03, 95% CI: 1.00–1.07), lack of appetite (OR: 1.09, 95% CI: 1.06–1.12), pain (OR: 1.08, 95% CI: 1.05–1.11), and poor well-being (OR: 1.05, 95% CI: 1.01–1.09) scores was associated with higher urgent healthcare use, after adjustment. We computed a global ESAS score using the highest individual symptom score (high-ESAS) for each assessment. Each 1-point increase in high-ESAS was associated with a 21% increase in the odds of urgent healthcare use (OR: 1.21, 95% CI: 1.18–1.24).

**Conclusion:**

ESAS scores are associated with subsequent short-term urgent healthcare use after NET diagnosis. This indicates a potential gap in managing outpatient patient-reported symptoms. Routine monitoring of ESAS scores could be leveraged to identify patients at high risk of urgent healthcare use in need for better symptom management.

## Introduction

Cancer diagnosis and treatment is associated with considerable symptom burden, which affects quality of life, receipt of care, and outcomes. Neuroendocrine tumors (NETs) represent a unique malignancy combining long-term survival with active disease and potentially debilitating symptoms from both tumor burden and hormonal secretion (1). Symptom tracking and ongoing supportive care are a challenge for patients with NETs. Using population-level data, we previously reported a high burden of patient-reported symptoms at the time of NET diagnosis that persists up to five years later (2). Predominant symptoms included tiredness, impaired well-being, and anxiety, impacting 30–45% of patients in the long term. Patients remained at risk of prolonged symptom burden over the years following diagnosis, indicating potential gaps in the management of patient-reported symptoms.

While randomized controlled trials have shown that routine screening with patient-reported outcome measures (PROMs) improves patient satisfaction, quality of life, use of emergency department (ED) visits, and overall survival, whether such benefits exist in real-world clinical settings is unclear (3, 4, 5, 6, 7, 8, 9). Over 10 years after the implementation of routine system-wide screening using the Edmonton Symptom Assessment System (ESAS) in Ontario, Canada, few healthcare providers indicated looking at PROM scores and integrating them in practice, and few patients received targeted intervention following reporting of high symptom scores (7, 8, 9, 10). The low rate of intervention after reporting of high symptom scores is multifactorial. The lack of understanding about the link between PROMs and patient outcomes, experience, and unaddressed needs in the real world are likely contributing (*what happens to patients who report high symptom scores?*).

Therefore, we leveraged population-level data to examine the association between routinely collected PROMs and subsequent ED visits and unplanned hospitalizations after a diagnosis of NETs, with a view to inform better support strategies for patients.

## Methods

### Study design

Through ICES (formerly known as the Institute for Clinical Evaluative Sciences), linked administrative healthcare datasets from the province of Ontario, Canada, were used to conduct a retrospective cohort study. The study was approved by the Research Ethics Board at Sunnybrook Health Sciences Centre and reported following the REporting of studies Conducted using Observational Routinely collected health Data (RECORD) statement (11).

### Study population

Individuals with valid Ontario Health Insurance Plan (OHIP) insurance from 2010 to 2019 were eligible for inclusion in the study. Ontario’s 16 million residents benefit from universally accessible and publicly funded healthcare through OHIP. Persons ≥18 years old with a new NET diagnosis between January 1, 2010, and March 31, 2019, were identified using International Classification of Diseases (ICD) codes using a strategy previously reported by our team (Supplementary Table 1 (see section on Supplementary materials given at the end of the article)) (12, 13, 14). Individuals were excluded if they were over the age of 105 years (routine data cleaning for the datasets used), had a prior diagnosis of cancer, had a subsequent cancer diagnosis within one year of their NET diagnosis, or had a date of death recorded prior to the date of diagnosis. Finally, those who completed at least one ESAS assessment within 24 months of NET diagnosis were retained for this cohort.

### Data sources

The OCR is a provincial database comprised of all patients with a cancer diagnosis (excluding non-melanoma skin cancers) since 1964 (15, 16). The Registered Persons Database (RPDB) contains vital status and demographic data on all individuals covered under the OHIP. Information regarding health services provided is included in the Canadian Institute for Health Information Discharge Abstract Database (CIHI-DAD), the National Ambulatory Care Reporting System (NACRS), the Cancer Activity Level Reporting, the OHIP Claims Database, and the Ontario Mental Health Reporting System (OMHRS) (17). The Ontario Laboratories Information System (OLIS) database contains information on tests performed in participating community, hospital, and public health laboratories. Datasets are detailed in Supplementary Table 2. The datasets were linked using a unique encoded identifier and analyzed at ICES.

### Index dates

**Figure 1:**
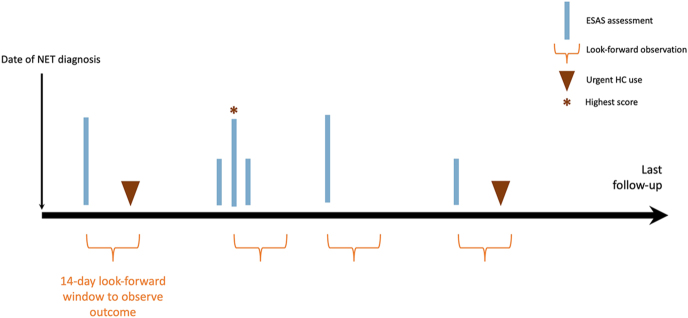
Timeline of exposure and outcomes measures. If more than one ESAS assessment happened in the same 14-day window, the one with the highest individual symptoms score was selected (tall vertical bar with asterisk vs small vertical bar).

Patients were observed from the date of NET diagnosis to the date of death, date of last contact, or end of study date (March 31, 2020), whichever came first. All ESAS scores reported in the 24 months following NET diagnosis were captured. If more than one ESAS score was identified during the same 14-day interval, the record with the highest scores was retained. If more than one ESAS score during the same 14-day interval had the same scores, the earlier one was retained. Each ESAS assessment was considered a separate index event, and we looked for the outcome of interest from each index date (Fig. 1).

### Outcomes

The outcome of interest was urgent healthcare utilization defined as a composite of ED visit and/or unplanned hospitalization. The outcome was captured within the 14-day window following each ESAS assessment. If an ED visit led to hospitalization, it was counted as a single hospitalization. We selected the 14-day window *a priori* based on clinical reasoning and prior work on other cancer sites (18). This window allows us to realistically correlate the symptom burden at the time of ESAS assessment to the healthcare episode while accounting for possible delays in seeking care due to initial self-management by patients (19, 20).

### Exposure

The exposure of interest was ESAS score at each index date. The ESAS score is a patient-reported outcome assessing the severity of nine common cancer-associated symptoms: pain, tiredness, drowsiness, nausea, lack of appetite, shortness of breath, depression, anxiety, and overall well-being. Its validity and reliability have been demonstrated in cancer populations (10, 11). Since 2007, ESAS scores are routinely collected at outpatient cancer visits across Ontario, using a numerical scale ranging from zero (no symptoms) to ten (worst possible symptoms). We examined ESAS scores in three ways: i) individual symptom scores, ii) the highest individual ESAS item (h-ESAS) defined as the highest individual symptom out of all nine symptoms, and iii) the total summed ESAS score (t-ESAS) defined as the sum of all individual symptom scores (0–90). Individual symptoms and highest individual ESAS item (h-ESAS) scores were categorized as mild (0–3), moderate (4–6), and severe (7–10), as previously described and validated (2, 18, 21, 22, 23). The t-ESAS was treated as a categorical variable by dividing it into 10-unit increments (18).

### Covariates

Baseline characteristics were measured at the time of NET diagnosis. Certain characteristics changed over time (such as age, comorbidity, or treatment category) and were updated at each index ESAS assessment for inclusion in multivariable models. Age and sex were abstracted from the RPDB. Rural residence was defined according to the Rurality Index of Ontario (24). Material deprivation quintile, a multi-dimensional, community-level measure incorporating socioeconomic factors such as education and income, assessed the socioeconomic status (25). Baseline comorbidity burden was measured using the Johns Hopkins Adjusted Clinical Groups system score based on healthcare services use with a 24-month look-back window prior to the date of NET diagnosis (26). The aggregated diagnosis groups (ADGs) were summed to create a total score and then dichotomized with a cutoff of 10 for high comorbidity burden (26, 27). Metastatic disease was identified with ICD-10 codes using a previously published algorithm and reported as synchronous (≤6 months from NET diagnosis) or metachronous (>6 months from NET diagnosis) (28). Treatment was categorized as surgery (primary tumor or liver resection), chemotherapy, and radiation therapy. Of note, peptide receptor radionuclide therapy (PRRT) was not available in Ontario over the study period, and information on somatostatin analogs was not available for the entire cohort within the available datasets. Treatment received in the 60 days prior to the ESAS assessment was used for multivariable models.

Finally, we captured measures of 24 h urinary 5-hydroxyindoleacetic acid (u5HIAA). We used OLIS to capture 24 h u5HIAA values from the date of NET diagnosis to the end of follow-up. For inclusion in the multivariable models, 24 h u5HIAA captured from NET diagnosis to the index ESAS assessment date was used. 24 h u5HIAA was reported as elevated or not, which accounted for variations in units and normal range values between laboratories. If no 24 h u5HIAA was available over the time window, it was considered missing.

### Statistical analysis

Baseline patient characteristics were described using absolute number (*n*) and percentage (%) for categorical variables, and median with interquartile range (IQR) for continuous variables. We initially compared the characteristics of patients who did and did not report ESAS scores using standardized differences, with differences >10% considered significant (29, 30).

We examined the association between ESAS scores and urgent healthcare utilization in the 14 days following ESAS completion using a series of logistic regression models. Three pairs of models (unadjusted and adjusted) were constructed, with the ESAS exposure modified for each pair of models. The first model included each of the nine individual symptom categorical scores as the exposure; all symptoms were included in the same model. The second and third models used categorical h-ESAS and t-ESAS as the exposure, respectively. Covariates adjusted for in multivariable models were defined *a priori* based on clinical relevance and the existing literature as potential confounders of the relationship between ESAS scores and urgent healthcare utilization (31, 32): age (continuous), sex, rural residence, material deprivation quintile (continuous, by quintile), primary tumor site, metastatic status, year of diagnosis (continuous, by 1-year increment), and treatment received in 60 days prior to ESAS assessment. We assessed collinearity using variance inflation factor with a cutoff of 2.5. A generalized estimating equation with an exchangeable correlation structure was used to account for clustering of ESAS scores at the patient level (33). Results are reported as odds ratio (OR) with 95% confidence interval (95% CI).

We examined missing data. Data were missing for rural residency in 1.1% and material deprivation in 0.5% of the cohort. Considering the high proportion of complete cases on those variables (99%), we performed a complete case analysis for those covariates. Data were missing for elevated 24 h u5HIAA in 52.6% of patients, because it was either unmeasured or unavailable due to the rollout of the OLIS database toward the end of the study time period. A missing category was created, and this covariate was not used in the primary analysis. We conducted a sensitivity analysis restricted to ESAS assessments with measured 24 h u5HIAA between the date of NET diagnosis and ESAS assessment whereby that covariate was added to adjusted models.

All analyses were two-sided, with statistical significance set at *P* < 0.05. Analyses were conducted using SAS Enterprise Guide 7.1 (SAS Institute, USA).

## Results

Of 8,514 patients diagnosed with NETs over the study period, 4,278 reported at least one ESAS score in the 24 months following NET diagnosis and were included in the study. The characteristics of included patients at the time of the first ESAS assessment are presented in Table 1. Patients who did and did not report at least one ESAS score in the 24 months after diagnosis were compared (Supplementary Table 3). Patients reporting ESAS scores were more likely to have been diagnosed with a small bowel or pancreas primary tumor and less likely to have a large bowel or rectum primary tumor. They were also more likely to have metastases and elevated 24 h u5HIAA.

**Table 1:** Characteristics of the entire cohort of patients with neuroendocrine tumors who completed at least one ESAS assessment in the two years after diagnosis.

		Patients
Characteristic*		(*n* = 4,278)
Age at diagnosis (years old), median (IQR)		63 (53–72)
Sex	Female	2,104 (49.2%)
	Male	2,174 (50.8%)
Rural residence		371 (8.7%)
Material deprivation quintile	1st (least deprived)	947 (22.1%)
	2nd	886 (20.7%)
	3rd	811 (19.0%)
	4th	813 (19.0%)
	5th (most deprived)	821 (19.2%)
High comorbidity burden (ADG ≥ 10)		1,370 (32.0%)
Primary tumor site	Stomach	209 (4.9%)
	Small bowel	1,083 (25.3%)
	Large bowel	492 (11.5%)
	Rectum	271 (6.3%)
	Pancreas	800 (18.7%)
	Lung	916 (21.4%)
	Others	507 (11.9%)
Metastatic status	None	2,090 (48.9%)
	Synchronous	1,633 (38.2%)
	Metachronous	555 (13.0%)
Elevated 24 h u5HIAA	Yes	1,317 (30.8%)
	No	712 (16.6%)
	Missing	2,249 (52.6%)
Treatment received after diagnosis	Surgery: primary tumor	2,193 (51.3%)
	Surgery: liver resection	266 (6.2%)
	Liver embolization	318 (7.4%)
	Chemotherapy	39 (0.9%)
	Radiation therapy	843 (19.7%)

Values are *n* (%) unless specified otherwise.

IQR, interquartile range; u5HIAA, urinary 5-hydroxyindoleacetic-acid.

* Characteristics presented at the time of first ESAS assessment; each covariate was updated at the time of index ESAS assessment for regression models.

**Figure 2:**
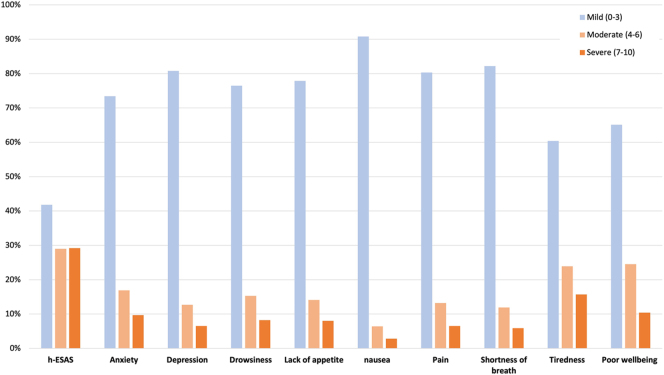
Distribution of ESAS scores in the entire cohort (*n* = 19,612 assessments).

A total of 19,612 ESAS scores were reported by included patients, with a median of 4 ESAS scores per patient (IQR: 2–7) in the 24 months after diagnosis. The distribution of ESAS scores is depicted in Fig. 2. The median t-ESAS was 13 (IQR: 5–27) out of 90 points. For individual symptom scores, tiredness, poor well-being, and anxiety had the highest median scores and the highest proportion of patients reporting moderate and severe scores. Overall, there were 891 ED visits without hospitalization, 478 ED visits followed by hospitalization, and 111 hospitalizations with no preceding ED visit, within 14 days of an ESAS assessment. The total number of events for the primary outcome for an ED visit and/or unplanned hospitalization within 14 days of an ESAS assessment was 1,480.

**Figure 3:**
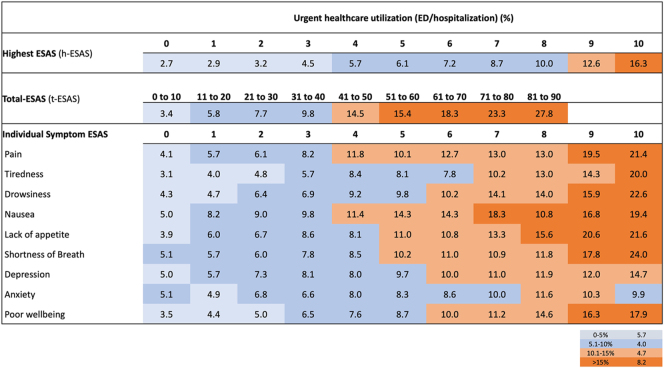
Distribution of patients with urgent healthcare utilization (ED or hospitalization) within 14 days of ESAS assessment, stratified by ESAS score.

The proportion of patients with ED visits and/or unplanned hospitalization in the 14 days after reporting an ESAS score varied depending on the individual, highest, and total ESAS scores (Fig. 3). On univariable analysis, reporting of moderate individual symptom scores of lack of appetite, pain, and tiredness and severe scores of drowsiness, lack of appetite, pain, tiredness, and poor well-being was associated with increased odds of 14-day ED visits and/or unplanned hospitalization. Reporting of moderate and severe h-ESAS was also associated with increased odds of 14-day ED visits and/or unplanned hospitalization, as was each 10-point increment in t-ESAS.

**Figure 4:**
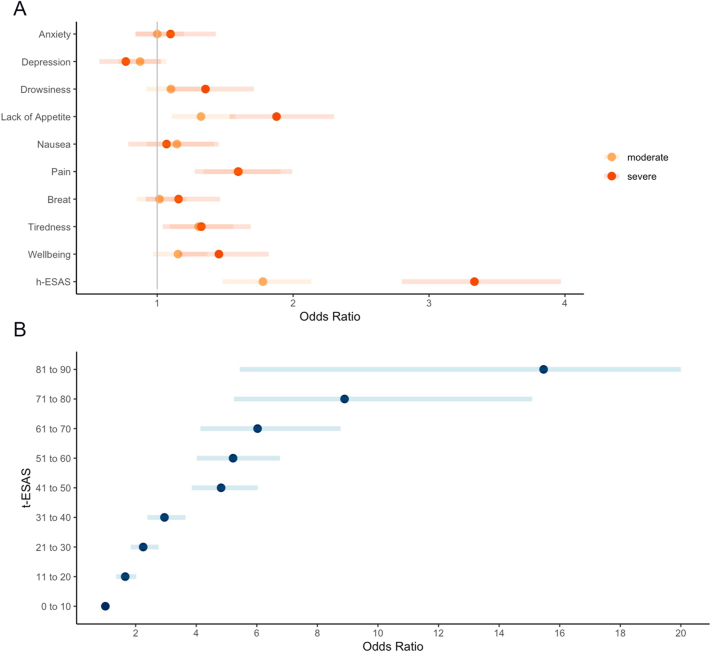
Adjusted odds of urgent healthcare utilization for individual symptom ESAS scores (A) and the total ESAS score (B). *Adjusted for patient’s age, sex, rural residence, material deprivation, primary tumor site, metastatic status, year of diagnosis, and treatment received in 60 days prior to ESAS assessment.

Adjusted associations between ESAS scores and 14-day ED visits and/or unplanned hospitalization are depicted in Fig. 4. Interactions between ESAS score and patient sex (*P* = 0.36 for h-ESAS) as well as between ESAS score and cancer site (*P* = 0.73 for h-ESAS) were explored and not significant. After adjusting for age, sex, rural residence, material deprivation quintile, primary NET site, metastatic status, year of diagnosis, and the type of treatment received in the 60 days prior to ESAS assessment, reporting of severe drowsiness and poor well-being and reporting of moderate to severe lack of appetite, pain, and tiredness were independently associated with increased odds of 14-day ED visits and/or unplanned hospitalization, compared to reporting of no or mild scores. Similarly, reporting of moderate and severe h-ESAS was independently associated with high odds of 14-day ED visits and/or unplanned hospitalization. Finally, when examining t-ESAS, each 10-point category was independently associated with increasing odds of 14-day ED visits and/or unplanned hospitalization, compared to a t-ESAS of 0–10. There was a dose–response relationship observed, with adjusted ORs going from 1.65 (95% CI: 1.36–2.02) for t-ESAS 11–20 to 15.5 (95% CI: 5.44–44.0) for t-ESAS 81–90, compared to 0–10.

In a sensitivity analysis restricted to index ESAS assessments with measured 24 h u5HIAA and adjusting for that variable, the associations observed in the entire cohort persisted (Supplementary Table 4).

## Discussion

In this population-level analysis of over 4,200 patients with NETs, we reported that patient-reported symptoms, as captured by ESAS scores, are independently associated with subsequent 14-day urgent healthcare use, as measured by ED visits and/or unplanned hospitalizations. After adjusting for key measured confounders, drowsiness, lack of appetite, pain, tiredness, and lack of well-being were independently associated with 14-day urgent healthcare use. The highest ESAS symptom score was associated with a 1.8-fold increase in the odds of 14-day urgent healthcare use if moderate and with a threefold increase in the same if severe. Finally, each increment of 10 points in the total ESAS score was associated with increased odds of 14-day urgent healthcare use, with a dose–response relation increased in those odds going from 1.6-fold for t-ESAS 11–20 to 15-fold for t-ESAS 81–90, compared to t-ESAS of 0–10. These findings are important because they outline a gap in the management of symptoms for patients with NETs.

Our findings contribute to the growing body of literature on patient-reported symptom burden in NETs. Previous research has examined patient-reported symptoms, captured by the validated ESAS score, across various cancers (34, 35, 36, 37, 38, 39, 40). Specifically for NETs, our team previously documented symptom patterns following diagnosis and at the end-of-life, identifying a persistently high burden of symptoms even five years after diagnosis (2, 41). The enduring symptom burden after diagnosis underscores the importance of addressing patient-reported symptoms in the management of NETs. Other studies have highlighted the high symptom burden in NETs and have pointed to the need for more evidence on integrating symptom information into practice and developing effective symptom management strategies (42). Looking at how patient-reported symptoms relate to healthcare needs, the association between the first ESAS score after diagnosis and ED visits within 7 days was previously examined in a heterogeneous population of all diagnosed cancers (43, 44). Members of our team have also examined patients with head and neck cancers and reported an association between ESAS scores and 14-day urgent healthcare utilization (18).

Our study builds on these findings by focusing specifically on NETs. This was an important population to target because it is particularly complex. Patients often live with the disease for extended periods, report considerable impacts on their quality of life due to symptom burden, and have expressed lacking clear care pathways and supportive care (45, 46, 47). NETs also represent a high burden for health systems with high costs and resource consumption (14, 48, 49, 50). The analysis herein leveraged the robust methodology described by Noel *et al.* for head and neck cancers to offer a detailed analysis of how patient-reported symptoms relate to urgent healthcare use in a large population of patients with NETs. The results provided actionable insights for clinical practice. In particular, they emphasized the need for a systematic integration of symptom screening and management into NET care pathways to improve patient experience and outcomes and optimize healthcare utilization.

While the benefits of patient-reported symptom screening, particularly using ESAS, are well documented in cancer care, these benefits are often not fully realized in everyday clinical practice. Randomized controlled trials have shown that symptom monitoring using ESAS can lead to improvements in quality of life, treatment adherence, and even overall survival, compared to usual care (4, 5, 51, 52, 53). However, translating these benefits from randomized controlled trials to real-world practice has proven challenging. Symptom monitoring alone may have limited impact if high symptom scores are not promptly followed by targeted interventions. For instance, prior work has indicated that less than 20% of patients reporting high depression scores on ESAS received psychosocial support and less than 3% received psychiatry assessment, and less than one-third of patients with high pain scores were prescribed pain medication (9, 54, 55).

Several barriers can hinder the effective use of patient-reported symptom scores in clinical practice, as documented in a survey of oncology practitioners (7). First, symptoms captured in general screening may not feel specific enough to be actionable in the context of certain cancer types. Second, reviewing these scores can be time-consuming. Finally, there is some uncertainty regarding the clinical implications of high scores, making it challenging for healthcare providers to decide on appropriate interventions. The current study reported increased odds in urgent healthcare use associated with ESAS scores that indicates both the value of ESAS in identifying patient needing care and a gap in receipt of care. Even if the symptoms assessed in ESAS were not specific to NETs, they were strongly associated with subsequent seeking of medical attention. Those results outline a need to better address reporting of high symptom scores by patients with NETs. Interventions to flag higher-risk patients for healthcare professionals may help in busy care environments and with strained resources. Predictive risk models have been suggested for this and garnered much attention in oncology recently (56, 57, 58, 59, 60). Such models could be developed for NETs, properly validated, and integrated into care pathways or electronic medical records to facilitate timely interventions.

This study has limitations. Due to the retrospective design and use of routinely collected data, the variables were not collected for the purpose of answering these specific research questions. This results in risk of misclassification bias and unmeasured confounding. Some information was not available, such as a detailed description of symptoms or the specific reasons for ED visits and unplanned hospitalizations. We also did not have information about all endocrine syndromes and, as such, could only assess this through a subgroup analysis of carcinoid syndrome (elevation in u5HIAA). With this study design, we also could not establish a causal relationship between high ESAS scores and urgent healthcare utilization. However, the temporality, specificity, plausibility, and the observed dose–response relationships make such a relationship likely (61). Another important consideration is the capture of ESAS scores that were only captured at outpatient visits and are known to be completed by certain groups of patients more than others (62). We reported the characteristics of patients who did and did not have available ESAS data over the study period, so this potential bias can be appreciated.

## Conclusions

Patient-reported symptoms, as captured by ESAS scores, were associated with subsequent short-term urgent healthcare use in patients with NETs. This indicates a potential gap in the care of NETs, specifically in managing high symptom scores. Care pathways and targeted interventions are needed to leverage routine monitoring of patient-reported symptoms to identify patients at high risk of urgent healthcare use in need for better symptom management.

## Supplementary materials



## Declaration of interest

JH received speaking honoraria from Ipsen.

## Funding

This work was funded by the North American Neuroendocrine Tumor Society Clinical Investigator Scholarship and an operating grant from the Canadian Institutes of Health Researchhttps://doi.org/10.13039/501100000024 (FRN #407301).

## Data availability

The datasets from this study are held securely in coded form at ICES. While data sharing agreements prohibit ICES from making the datasets publicly available, access may be granted to those who meet pre-specified criteria for confidential access, available at https://www.ices.on.ca/use-ices-data/. The dataset creation plan and underlying analytic code are available from the authors upon reasonable request, on the understanding that the computer programs may rely upon coding templates or macros that are unique to ICES and are therefore either inaccessible or may require modification.
