# Supplementary material for: Patient-reported symptom and urgent healthcare use in neuroendocrine tumors
Source: Endocr Oncol. 2026 Jan 19;6(1):e250050. doi: 10.1530/EO-25-0050 (PMC12821087; doi:10.1530/EO-25-0050)
Supplement: Supplementary file 1 [file supplementary_materials.pdf]

__**ONLINE ONLY SUPPLEMENT**__

eTable 1. Coding strategy used to identify patients diagnosed with neuroendocrine tumors and determine tumor characteristics.

eTable 2. Data Sources

eTable 3. Characteristics of patients diagnosed with neuroendocrine tumor who did and did not complete ESAS assessments in the two years after diagnosis (captured at the time of NET diagnosis).

eTable 4. Unadjusted and adjusted* odds ratios of urgent healthcare utilization by ESAS scores.

eTable 5. Unadjusted and adjusted odds ratios of urgent healthcare utilization by ESAS scores, for the sensitivity analysis adjusting for 24-hour urinary 5HIAA (n= 4,537 assessments)**.**

eTable 1. Coding strategy used to identify patients diagnosed with neuroendocrine tumors and determine tumor characteristics.

1. **Identify neuroendocrine tumors**

ICD-O.3

81503	Islet cell carcinoma
81501	Islet cell tumor, NOS
81510	Insulinoma, NOS
81513	Insulinoma, malignant
81521	Glucagonoma
81523	Glucagonoma, malignant
815*31*	Gastrinoma
81533	Gastrinoma, malignant
81543	Mixed islet-cell/exocrine adenocarcinoma
81551	VIPoma
81553	VIPoma, malignany
81561	Somatostatinoma
81563	Somastatinoma, Malignant
81571	Enteroglucagonoma
81573	Entroglucagonomam, malignant
82401	Carcinoid tumor of uncertain malignant potential
82403	Carcinoid tymor, nos
82413	Enterochromaffin-like Cell Carcinoid
82421	Enterochromaffin-like cell tumours, nos
82423	Enterochromaffin-like cell tumours, malignant
82443	Composite carcinoid
82453	Adenocarcinoid
82451	Tubular carcinoid
82463	Neuroendocrine carcinoma
82493	Atypical carcinoid

Exclusions

ICD-O.3

80023	Malignant tumour, small cell type
80400	Tumorlet
80413	Small cell carcinoma NOS
80423	Oat Cell carcinoma
80433	Small cell carcinoma NOS, fusiform cell type
80443	Small cell carcinoma, intermediate cell
80453	Combined small cell carcinoma
80133	Large cell neuroendocrine carcinoma of the lung
87000	Pheochromocytoma, nos
87003	Pheochromcytoma, maligangt
86801	Paraganglioma
86803	Paraganglioma, malignant
86931	Extra-adrenal paraganglioma
86933	Extra-adrenal paraganglioma, malignant
85103	Medullary carcinoma of the thyroid
80000	Neroplasm, benign
80001	Neoplasm, uncertain whether benign or malignant

80003	Neoplasm, malignant
80100	Epithelial tumor, benign
80702	Squamouse cell carcinoma, in situ, ons
80703	Squamous, cell carcinoma, nos
80706	Squamous cell carcinoma, metastatic nos
81400	Adenoma, nos
81401	Atypical adenoma
83413	Papillary microcarcinoma
84813	Mucinous producing adenocarcinoma
85002	Intraductal carcinoma, non infiltrating, nos
85003	Infiltrating duct carcinoma, nos
93643	Peripheral neuroectodermal tumor
93703	chordoma
82433	Goblet Cell Carcinoid

2. **Define primary tumor type**

Topography codes

- Stomach, ICD-O.3: C16*
- Small Intestine, ICD-O.3: C17*
- Large Intestine, ICD-O.3: C18*, C19*
- Rectum, ICD-O.3: C20*
- Pancreas, ICD-O.3: C25*
- Bronchus, ICD-O.3: C34*

Others: all others

eTable 2. Data Sources

Database	Description
**Ontario Cancer Registry (OCR)**	The OCR is a passive, provincial registry all incident cancer diagnoses in Ontario. It includes 96% of cancer diagnoses in the province. Information included in the registry: cancer topography and morphology/histology, and details on diagnosis (e.g., types of contributing information to the diagnosis, dates).
**Activity Level Report (ALR) of Cancer Care Ontario**	Cancer Care Ontario maintains a database of cancers-specific services, including consultations, chemotherapy, and radiotherapy provided by regional cancer centers in the province. Because all radiotherapy is delivered at RCCs, it is a complete source of information. However, because not all cancer patients who receive surgery or chemotherapy visit an RCC, it cannot be used as a population data sources for those treatments.
**Registered Patient Database (RPDB)**	The RPDB is an ICES database derived from all administrative data sources and provides demographic data including age, patient residence, vital status, date of last contact with the healthcare system, and OHIP eligibility.
**Ontario Marginalization Index (ONMarg)**	The ON-MARG is a specialized database using Census data to profile relative area-level marginalization dependency, deprivation, ethnic concentration, and instability at various geographic levels in Ontario.
**Ontario Health Insurance Plan (OHIP)**	The OHIP database contains all physician billing data including information on diagnoses as well as services provided, such as receipt of surgery, chemotherapy, and radiotherapy.
**Ontario Drug Benefit Database (ODB)**	The ODB contains all information for prescription drug claims for individuals covered by the provincial plan (all individuals $\geq$65 years old), including type of medication, dose, date of prescription filling, and duration of treatment.
**Canadian Institute of Health Information – Discharge Abstract Database and Same-Day Surgery (CIHI-DAD and SDS)**	CIHI-DAD and SDS are mandatory reporting systems that provide information on hospital admissions and same-day surgeries, including diagnoses, procedures, and length of stay.
**Symptoms monitoring and reporting database (SMRD)**	Edmonton Symptom Assessment System scores reported at outpatient cancer visits.

eTable 3. Characteristics of patients diagnosed with neuroendocrine tumor who did and did not complete ESAS assessments in the two years after diagnosis (captured at the time of NET diagnosis).

**Characteristic**		**No ESAS reported** (n=4,236)	**≥1 ESAS reported** (n=4,278)	**Standardized difference** (%)*
**Age at diagnosis** (years old)	Median (IQR)	61 (50-71)	63 (53-72)	10
**Sex**	Female	2,235 (52.8%)	2,104 (49.2%)	7
	Male	2,001 (47.2%)	2,174 (50.8%)	7
**Rural residence**	Yes	3,848 (90.8%)	3,862 (90.3%)	2
	No	340 (8.0%)	371 (8.7%)	2
	Missing	48 (1.1%)	45 (1.1%)	1
**Material deprivation quintile**	1st (least deprived)	813 (19.2%)	924 (21.6%)	6
	2nd	808 (19.1%)	886 (20.7%)	4
	3rd	824 (19.5%)	811 (19.0%)	1
	4th	873 (20.6%)	813 (19.0%)	4
	5th (most deprived)	881 (20.8%)	821 (19.2%)	4
	Missing	37 (0.9%)	23 (0.5%)	4
**High comorbidity burden** (ADG ≥10)	Yes	1,338 (31.6%)	1,370 (32.0%)	1
	No	2,898 (68.4%)	2,908 (68.0%)	1
**Primary tumor site**	Large bowel	792 (18.7%)	492 (11.5%)	20
	Lung	966 (22.8%)	916 (21.4%)	3
	Other	325 (7.7%)	507 (11.9%)	14
	Pancreas	323 (7.6%)	800 (18.7%)	33
	Rectum	984 (23.2%)	271 (6.3%)	49
	Small bowel	547 (12.9%)	1,083 (25.3%)	32
	Stomach	299 (7.1%)	209 (4.9%)	9
**Metastatic status**	None	3,278 (77.4%)	2,090 (48.9%)	62
	Synchronous	810 (19.1%)	1,633 (38.2%)	43
	Metachronous	148 (3.5%)	555 (13.0%)	35
**Elevated 24-hour urinary 5HIAA**	Yes	387 (9.1%)	1,317 (30.8%)	56
	No	499 (11.8%)	712 (16.6%)	14
	Missing	3,350 (79.1%)	2,249 (52.6%)	58
**Year of diagnosis**	2010	402 (9.5%)	247 (5.8%)	14
	2011	421 (9.9%)	316 (7.4%)	9
	2012	398 (9.4%)	396 (9.3%)	0
	2013	433 (10.2%)	462 (10.8%)	2
	2014	454 (10.7%)	494 (11.5%)	3
	2015	511 (12.1%)	530 (12.4%)	1
	2016	475 (11.2%)	612 (14.3%)	9
	2017	519 (12.3%)	570 (13.3%)	3
	2018	542 (12.8%)	562 (13.1%)	1
	2019	81 (1.9%)	89 (2.1%)	1

Values are reported as n (%), unless specified otherwise.
*>10% considered a significant difference

eTable 4. Unadjusted and adjusted odds ratios of urgent healthcare utilization by ESAS scores, for the sensitivity analysis adjusting for 24-hour urinary 5HIAA (n= 4,537 assessments)**.**

ESAS score			Unadjusted Odds Ratio (95%CI)	Adjusted Odds Ratios (95%CI)
**Total ESAS score** (reference: 0-10)		11-20	1.97(1.32-2.92)	1.97 (1.32-2.92)
		21-30	1.98 (1.26-3.10)	1.98 (1.26-3.10)
		31-40	2.77 (1.75-4.39)	2.77 (1.75-4.39)
		41-50	4.45 (2.78-7.27)	4.50 (2.78-7.27)
		51-60	4.64 (2.65-8.12)	4.64 (2.65-8.12)
		61-70	6.93 (3.19-15.05)	6.93 (3.19-15.05)
		71-80	2.46 (0.35-17.14)	2.46 (0.35-17.14)
		81-90	31.20 (7.53-129.68)	31.25 (7.53-129.68)
**Highest ESAS score** (reference: mild – 0-3)		4 to 6	1.51 (1.05-2.12)	1.51 (1.05-2.19)
		7 to 10	2.66 (1.89-3.76)	2.66 (1.89-3.76)
**Individual symptoms ESAS scores** (reference: mild – 0-3)	**Anxiety**	Moderate (4-6)	0.638 (0.422-0.966)	0.74 (0.49-1.12)
		Severe (7-10)	0.974 (0.563-1.685)	1.24 (0.72-2.15)
	**Depression**	Moderate (4-6)	1.131 (0.718-1.781)	1.07 (0.68-1.67)
		Severe (7-10)	0.892 (0.452-1.759)	0.84 (0.43-1.63)
	**Drowsiness**	Moderate (4-6)	1.171 (0.753-1.819)	1.02 (0.67-1.56)
		Severe (7-10)	1.442 (0.798-2.606)	1.16 (0.63-2.12)
	**Lack of appetite**	Moderate (4-6)	1.15 (0.754-1.756)	1.11 (0.74-1.67)
		Severe (7-10)	1.567 (0.994-2.471)	1.49 (0.95-2.35)
	**Nausea**	Moderate (4-6)	1.192 (0.718-1.978)	1.33 (0.79-2.23)
		Severe (7-10)	1.066 (0.503-2.259)	1.14 (0.52-2.48)
	**Pain**	Moderate (4-6)	1.538 (1.044-2.264)	1.54 (1.05-2.26)
		Severe (7-10)	1.281 (0.781-2.101)	1.17 (0.71-1.92)
	**Dyspnea**	Moderate (4-6)	1.271 (0.875-1.847)	1.54 (1.05-2.26)
		Severe (7-10)	0.836 (0.455-1.538)	1.17 (0.7-1.92)
	**Tiredness**	Moderate (4-6)	1.112 (0.748-1.655)	1.12 (0.77-1.64)
		Severe (7-10)	1.261 (0.713-2.232)	1.32 (0.77-2.29)
	**Poor wellbeing**	Moderate (4-6)	1.458 (0.985-2.156)	1.43 (0.98-2.08)
		Severe (7-10)	1.67 (1.022-2.729)	1.78 (1.11-2.85)

*adjusted for adjusted for patient age, sex, rural residence, material deprivation, primary tumor site, metastatic status, year of diagnosis, treatment received in 60 days prior to ESAS assessment, and elevated 24-hour urinary 5HIAA prior to ESAS assessment.
95%CI: 95% confidence
